# Saturated Very Long Chain Fatty Acids Are Required for the Production of Infectious Human Cytomegalovirus Progeny

**DOI:** 10.1371/journal.ppat.1003333

**Published:** 2013-05-16

**Authors:** Emre Koyuncu, John G. Purdy, Joshua D. Rabinowitz, Thomas Shenk

**Affiliations:** 1 Department of Molecular Biology, Princeton University, Princeton, New Jersey, United States of America; 2 Department of Chemistry and Lewis Sigler Institute for Integrative Genomics, Princeton University, Princeton, New Jersey, United States of America; University of North Carolina at Chapel Hill, United States of America

## Abstract

Human cytomegalovirus hijacks host cell metabolism, increasing the flux of carbon from glucose to malonyl-CoA, the committed precursor to fatty acid synthesis and elongation. Inhibition of acetyl-CoA carboxylase blocks the production of progeny virus. To probe further the role of fatty acid metabolism during infection, we performed an siRNA screen to identify host cell metabolic enzymes needed for the production of infectious cytomegalovirus progeny. The screen predicted that multiple long chain acyl-CoA synthetases and fatty acid elongases are needed during infection, and the levels of RNAs encoding several of these enzymes were upregulated by the virus. Roles for acyl-CoA synthetases and elongases during infection were confirmed by using small molecule antagonists. Consistent with a role for these enzymes, mass spectrometry-based fatty acid analysis with^13^C-labeling revealed that malonyl-CoA is consumed by elongases to produce very long chain fatty acids, generating an approximately 8-fold increase in C26-C34 fatty acid tails in infected cells. The virion envelope was yet further enriched in C26-C34 saturated fatty acids, and elongase inhibitors caused the production of virions with lower levels of these fatty acids and markedly reduced infectivity. These results reveal a dependence of cytomegalovirus on very long chain fatty acid metabolism.

## Author Summary

Herpes viruses modulate cellular pathways to generate the building blocks that are necessary for their replication. Human cytomegalovirus alters metabolism of infected cells and causes a dramatic increase in lipid biosynthesis. We have investigated the role of lipid pathways in the viral life cycle and discovered that the virus requires several host enzymes that are responsible for the synthesis of very long chain fatty acids. Interestingly, very long chain fatty acids are substantially increased in the lipids of infected cells and saturated forms of these fatty acids are selectively incorporated into the envelope of the virus. Drugs that inhibit the synthesis of very long chain fatty acids generate virus particles with reduced infectivity. The discovery that human cytomegalovirus depends on the production of particular fatty acids furthers our understanding of virus-host cell interaction and suggests potential novel strategies for antiviral therapies.

## Introduction

Viruses reprogram host cell functions to facilitate their replication. Human cytomegalovirus (HCMV) profoundly alters cellular homeostasis, instituting its own metabolic program. HCMV infection up-regulates flux through much of central metabolism, at least in part through AMP kinase activation [1], [2]. A particularly strong flux increase involves the TCA cycle and its efflux to feed fatty acid metabolism [3], [4], [5], [6]. While AMP kinase is known to phosphorylate and thereby inhibit the committed enzyme of fatty acid synthesis, acetyl-CoA carboxylase (ACC), HCMV overrides this regulatory mechanism. Flux through ACC is markedly increased during infection and pharmacological or siRNA-mediated inhibition of ACC reduces the production of virus [4], [7].

Here we have investigated how HCMV utilizes the product of ACC, malonyl-CoA, which accumulates in HCMV-infected cells. To this end, we performed an siRNA screen to identify metabolic enzymes that contribute to viral growth. This screen, in combination with subsequent studies with small molecule enzyme inhibitors, identified an important role in the HCMV life cycle for long chain fatty acyl-CoA synthetases and fatty acid elongases. Both of these classes of enzymes contribute to the synthesis of lipids with long chain fatty acid (LCFA; 14–20 carbons) and very long chain fatty acid (VLCFA; ≥22 carbons) tails. Human long chain acyl-CoA synthetases include five acyl-CoA synthetase long-chain (ACSL) proteins and six solute carrier family 27 (SLC27A) proteins, all of which activate fatty acids to form acyl-CoAs, with the ACSL proteins generally acting on LCFA, and SLC27A proteins generally acting on VLCFA substrates [8], [9]. The activated fatty acids, which may come from diet, cellular stores, or fatty acid synthase, can then be used to make triglycerides, phospholipids, or energy. Moreover, these fatty acyl-CoAs can be elongated by the fatty acid elongases (ELOVLs), a class of seven different proteins in humans [10]. ELOVLs consume malonyl-CoA to add two-carbon units to fatty acyl-CoA substrates.

Consistent with the virus' dependence on ACC, ACSLs, SLC27As and ELOVLs, global analysis of saponified fatty acids revealed that HCMV-infected cells consume malonyl-CoA to elongate fatty acids into VLCFAs, producing virion envelopes strikingly enriched for saturated VLCFAs (C26:0-C34:0). ELOVL inhibition blocked accumulation of these VLCFAs, impaired production of virus particles, and markedly decreased the infectivity of those particles that were produced. The effect of ELOVL inhibition on the yield of infectious virus was overcome by feeding a saturated VLCFA. Collectively, these experiments reveal a striking dependence of HCMV infectivity on VLCFAs.

## Results

### Fatty acid metabolic enzymes modulate HCMV replication

**Figure 1:**
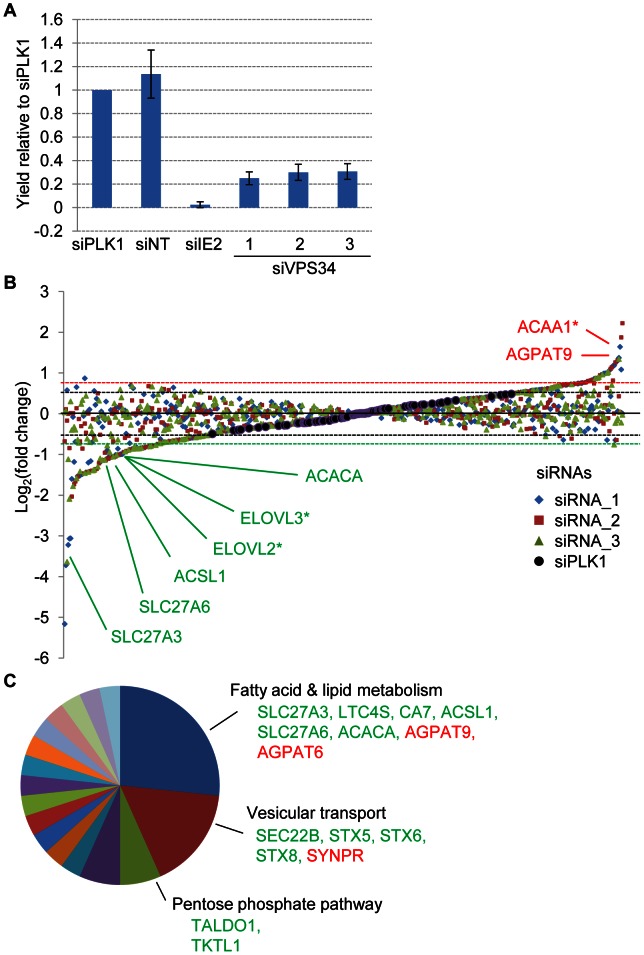
siRNA screen for cellular metabolic enzymes required for efficient HCMV replication. Fibroblasts were transfected in 96-well plates with siRNAs and mock-infected or infected with BAD*in*UL99GFP (0.5 IU/cell) 24 h later. HCMV yield in the supernatant was determined at 96 hpi. **(A)** Effect of siRNA controls used in the screen on HCMV yield. siRNAs targeting the cellular kinase PLK1, non-targeting siRNA (siNT), HCMV IE2 (siIE2) and the cellular kinase VPS34 (siVPS34_1–3) were included on each 96-well plate along with siRNAs to be screened for antiviral activity. The values represent the normalized yields from 18 independent experiments. siNT was assayed in octuplicate (n = 144) and siPLK1 was assayed in quadruplicate in each experiment (n = 72). Error bars represent ±1 standard deviation of the mean. **(B)** siRNAs targeting genes associated with fatty acid metabolism were identified as hits in the screen. Three siRNAs were tested for each target, and their effect on HCMV yield was quantified, log_2_-transformed and plotted. The siRNA showing the most robust effect on HCMV yield are identified for several enzymes. siRNAs designated in green type reduce virus yield and those in red type elevate virus production. Hits marked by an asterisk (*) are cases where only one siRNA out of three affected the virus yield. The dashed black horizontal lines delimit the spread of the 72 controls in the assay (1.4 times the mean) and the dashed colored horizontal lines mark the deviation from the control mean (3× SD) required to consider an siRNA a hit. **(C)** siRNA hits grouped by gene ontology analysis.

To identify host cell metabolic enzymes that are required for efficient production of HCMV progeny, we performed an siRNA screen targeting 401 cellular enzymes, including many involved in fatty acid and lipid metabolism (Figure S1A). Fibroblasts in 96-well plates were transfected with 3 different siRNAs for each target and 24 h later cells were infected with HCMV (0.5 IU/cell). Virus released into culture medium was assayed after an additional 96 h. This schedule was designed to produce the greatest possible knockdown of the cellular target during the course of the extended HCMV replication cycle. Each plate assayed included as negative controls non-targeting siRNAs and siRNAs specific for the host cell protein PLK1, which does not affect HCMV replication, and as positive controls siRNAs specific for the immediate early viral protein IE2 and the host cell protein VPS34, which is required for HCMV replication (R. Sharon-Friling and T. Shenk, unpublished) (Figure 1A). For each sample, the yield of virus was normalized to the average of internal negative controls (PLK1 siRNAs) after correction for edge effects, and the fold-change was log_2_-transformed (Figure 1B and Table S1). PLK1-specific siRNA was assayed 72 times during the screen at different positions in the 96-well plates and generated a standard deviation of <0.156 from the mean virus yield, which was 0.995. An siRNA was considered a hit if it altered the virus yield by >3 standard deviations relative to the mean of control siRNAs. Using the additional requirement that at least 2 out of 3 siRNAs against a target produced hits, we identified 26 enzymes (∼6.5% of the library) whose knockdown reduced virus yield and 6 enzymes (∼1.5% of the library) whose knockdown increased virus yield. Using the less stringent requirement of a single siRNA, which generates some false positives, we identified 65 additional enzymes whose knockdown reduced and 51 that increased virus yield. Gene ontology analysis [11], combined with literature searches, revealed that multiple hits fell into the category of fatty acid and lipid metabolism (Figure 1C). Four enzymes associated with the metabolism of fatty acids were required to support virus replication based on ≥2 siRNA hits: ACC alpha subunit (ACACA), ACSL1, and SLC27A3, and SLC27A6. While ACC is known to contribute to the production of HCMV progeny [4], [7], the other enzymes were not previously associated with HCMV replication. ACSL1, SLC27A3 and SCL27A6 consume ATP to ligate long and VLCFAs with coenzyme A (CoA) to make very long chain fatty acyl-CoAs. Knockdown of two other enzymes producing very long chain fatty acyl-CoAs, elongases ELOVL2 and ELOVL3, inhibited virus replication based on a single siRNA hit. In contrast, two of the proteins whose knockdown enhances the production of HCMV progeny consume very long chain fatty acyl CoAs: acetyl-CoA acyl-transferase 1 (ACAA1, a peroxisomal β-oxidation enzyme) and 1-acylglycerol-3-phosphate O-acyltransferase 9 (AGPAT9), which consumes fatty acyl-CoAs to form triglycerides [12]. Thus, the siRNA screen revealed that enzymes making VLCFAs promote viral replication and those consuming VLCFAs inhibit the pathogen.

### HCMV modulates the expression of genes involved in fatty acid metabolism

**Figure 2:**
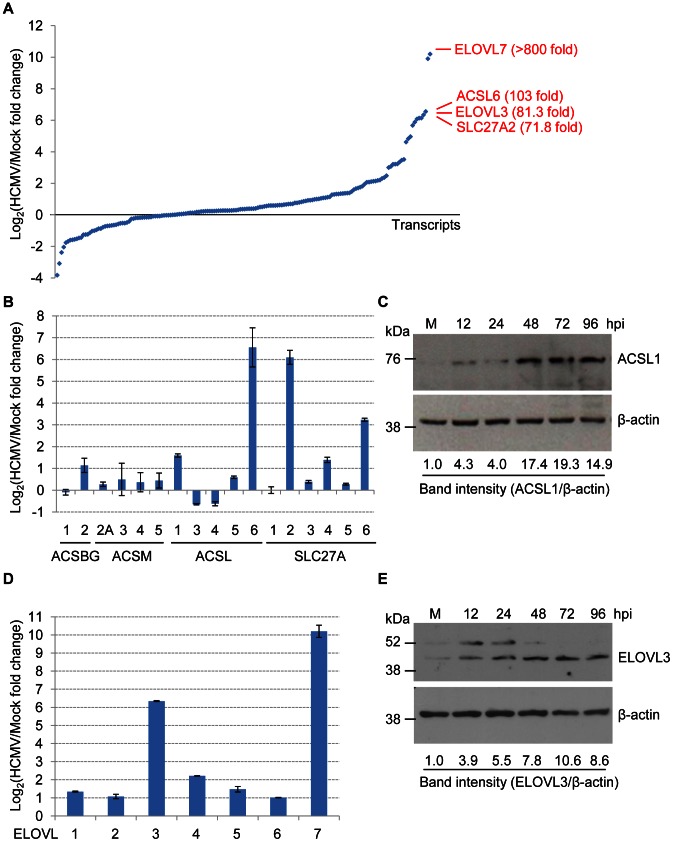
Identification of genes involved in fatty acid metabolism that are induced during HCMV infection. **(A)** RT-qPCR analysis of cellular RNAs. RNA was analyzed at 48 hpi with BAD*in*UL99GFP (10 IU/cell). Results were normalized to the expression level of 4 housekeeping genes, except in the case of elongases where GAPDH alone was used, and presented as log_2_-transformed fold change (infected/mock). Fold increases of ELOVL7, ACSL6, ELOVL3 and SLC27A2 are indicated. The results show the average of two independent experiments. **(B)** RT-qPCR analysis of acyl-CoA synthetase RNAs in HCMV-infected cells. Results are from data in panel A. Error bars represent ±1 SD of the mean. **(C)** ACSL1 protein accumulates during HCMV infection. Fibroblasts were mock-infected or infected with BAD*in*UL99GFP (3 IU/cell), harvested after indicated time intervals and analyzed by western blot by using antibody to ACSL1. β-actin was monitored as a loading control. Band intensities were quantified using ImageJ software and ratio of ACSL1/β-actin after infection was compared to that of mock infected cells (M). **(D)** RT-qPCR analysis of elongase RNAs. RNA was analyzed at 48 hpi with BAD*in*UL99GFP (10 IU/cell). Results are from two independent experiments assayed in triplicate. Error bars represent ±1 SD of the mean. **(E)** ELOVL3 protein accumulates in HCMV-infected cells. Cells were treated as in panel C and analyzed by western blot by using an antibody to ELOVL3. β-actin was monitored as a loading control. Band intensities were quantified as in panel C.

Since the siRNA screen predicted that enzymes sponsoring VLCFA metabolism play a critical role in the HCMV life-cycle, we investigated the effect of infection on their expression. PCR-arrays were used to quantify the levels of transcripts for 172 genes associated with fatty acid metabolism and adipogenesis, including the entire family of medium (ACSM2A, 3–5), long (ACSBG1–2, ACSL1, 3–6) and very long chain fatty acyl-CoA synthetases (SLC27A1–6), as well as all seven members of the elongase family (ELOVL1–7). RNA was assayed at 48 hpi, since dramatic changes in metabolite levels are observed at this time after infection [5]. Using a cut-off of a ≥3-fold change to identify altered transcripts, 37 RNAs (∼21.5%) were up-regulated, whereas 10 (∼5.8%) were down-regulated in response to infection (Figure 2A, and S2). Strikingly, four of the five most up-regulated RNAs encoded fatty acid-activating enzymes and elongases: ACSL6 and SLC27A2 increased by ∼103 and 72-fold, and ELOVL3 and ELOVL7 increased by ∼81 and >800-fold. Other up-regulated fatty acid-activating enzymes included ACLS1 and SLC27A6 (Figure 2B). Additional up-regulated elongases included ELOVL4, and, to a lesser extent, ELOVL1, 2 and 6 (Figure 2D). To confirm the transcript results, we analyzed the protein levels of two of the siRNA hits, ACSL1 and ELOVL3, during HCMV infection and found that both proteins were elevated (Figure 2C and E). The bands detected by ELOVL3 antibody migrate slower than the predicted 31 kDa size of ELOVL3, consistent with it being an N-glycosylated protein [13].

### Fatty acyl-CoA synthetase and elongase activities are required for the production of infectious HCMV progeny

**Figure 3:**
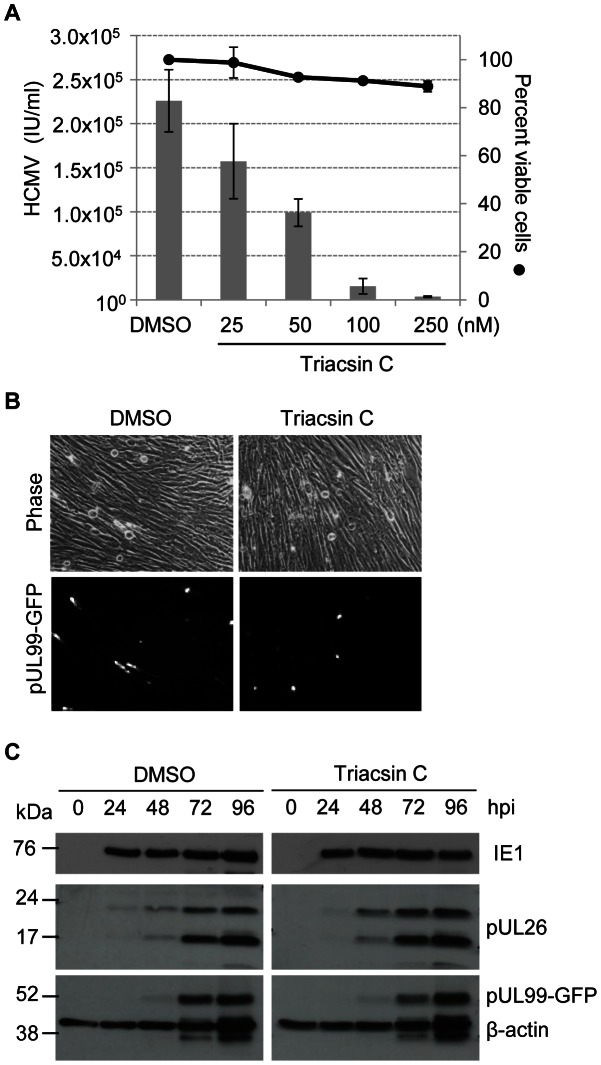
Triacsin C inhibits HCMV replication. **(A)** Triacsin C blocks the production of HCMV progeny. Fibroblasts were infected with BAD*in*UL99GFP (1 IU/cell) and maintained in medium containing 10% serum. Cultures were treated at 2 hpi with DMSO (vehicle) or triacsin C at indicated concentrations. At 96 hpi, cell viability and virus yield (intracellular plus extracellular) was assayed. The results are from two biological replicates. Error bars represent ±1 SD of the mean. **(B)** Triacsin C does not block HCMV cytoplathic effect or pUL99-GFP expression. Fibroblasts were infected with BAD*in*UL99GFP (1 IU/cell) and treated with triacsin C (250 nM) at 2 hpi. At 96 hpi images were captured by phase microscopy and fluorescent microscopy. **(C)** Triacsin C does not affect the accumulation of immediate early, early and late viral proteins. Fibroblasts were infected with BAD*in*UL99GFP (1 IU/cell) and treated with DMSO or triacsin C (250 nM) at 2 hpi. Cells were harvested at indicated times after infection and processed for western blot analysis using antibodies specific for IE1, pUL26 and pUL99. β-actin served as a loading control.

To verify the siRNA results, we analyzed the effect of small molecule inhibitors of fatty acid activation and elongation on HCMV replication. Triacsin C inhibits multiple ACSLs showing selectivity for ACSL1 and 4 [14], [15]. Fibroblasts were infected with HCMV in the presence of serum, treated with Triacsin C, and virus production was assayed at 96 hpi. Triacsin C inhibited the production of HCMV in a dose dependent manner without inducing cytotoxicity (Figure 3A). Antiviral effects occurred in the same dose range where triacsin is known to impact lipid metabolism [14], [16]. Triacsin C did not prevent HCMV's cytopathic effect (cell rounding) (Figure 3B) or expression of the immediate early IE1, early pUL26 or late pUL99 viral protein (Figure 3C). These observations suggest that the drug acts to block the production of virus after synthesis of the late protein, possibly during virion assembly.

**Figure 4:**
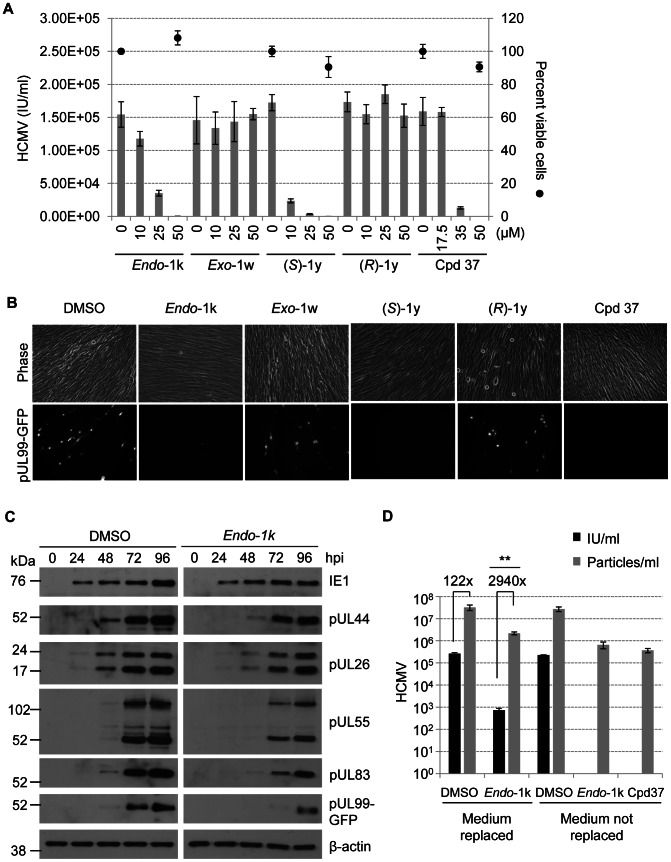
Pharmacological inhibitors of fatty acid elongases reduce HCMV yield. **(A)** Elongase inhibitors, but not inactive derivatives, block the production of infectious progeny. At 2 hpi with BAD*in*UL99GFP (1 IU/cell), fibroblasts were treated with DMSO (vehicle) or drugs at indicated concentrations. At 96 hpi, virus yield (intracellular plus extracellular) was assayed, and cell viability was monitored for indicated samples. The results report two biological replicates. Error bars represent ±1 SD of the mean. **(B)** Elongase inhibitors block cytopathic effect and pUL99-GFP accumulation. Cells were infected with BAD*in*UL99GFP (1 IU/cell) and treated with drugs (50 µM) at 2 hpi. At 96 hpi images were captured by phase microscopy and fluorescent microscopy. **(C)** Effect of *Endo*-1k on the accumulation of viral proteins. Fibroblasts were infected with BAD*in*UL99GFP (1 IU/cell) and treated with DMSO or *Endo*-1k (50 µM) at 2 hpi. Cells were harvested at indicated times after infection and processed for western blot analysis using antibodies specific for IE1, pUL44, pUL26, pUL55, pUL83 and pUL99. β-actin served as a loading control. **(D)** Elongase inhibitors reduce the infectivity of virions . Cells were treated with drugs (50 µM) at 2 hpi, and received fresh medium and drugs at 48 hpi (Medium replaced) or maintained without re-feeding (Medium not replaced). At 96 hpi, virus particles and infectivity (IU) were quantified. Results report three independent experiments, and error bars represent ±1 SD of the mean. **p<0.005 (t-test, ratio of virus particles/infectivity after drug treatment was compared to that of DMSO treated cells).

Next, we focused on elongases. Three structurally distinct ELOVL antagonists have been described. *Endo*-1*k* [17], *(S)*-1y [18], and Compound 37 [19] are potent inhibitors of ELOVL6 (IC_50_ = 3–80 nM), with weaker activity against ELOVL3 (IC_50_ = 0.2–7 µM), limited activity against ELOVL1, 2 and 5, and unknown effects on ELOVL4 and 7. All three compounds inhibited the production of infectious progeny in a dose-dependent manner, with micromolar concentrations required for the antiviral effects, suggesting that inhibition of additional elongases beyond ELOVL6 is required to block the production of infectious virus (Figure 4A). These compounds did not significantly affect the viability of mock- or HCMV-infected cells. The antiviral effects were stereospecific: *Exo*-1*k*, an inactive derivative of *Endo*-1*k*, and *(R)*-1y, an inactive stereoisomer of *(S)*-1y, did not influence the production of infectious virus. Intriguingly, we observed a loss of viral cytopathic effect and inhibition of pUL99 accumulation in cells treated with each of the elongase inhibitors (Figure 4B). Western blot analysis (Figure 4C) confirmed a substantial delay in accumulation of the true late protein, pUL99, in *Endo*-1k treated cells. The accumulation of a “leaky” late (pUL83), delayed early (pUL55) and early proteins (pUL26 and pUL44) were also delayed by the drug, but to a lesser extent. The immediate early protein, IE1, on the other hand, was unaffected. These results suggest that, in contrast to ACSL activity (Figure 3), elongase activity supports an aspect of viral replication prior to virion assembly. For example, newly synthesized VLCFAs might be needed to signal progression through early stages of viral replication, and readiness to advance to late protein synthesis.

ELOVL inhibitors could prevent the accumulation of infectious HCMV progeny by blocking the production of virions and/or by causing the production of particles with reduced infectivity. To distinguish between these possibilities, we assayed the particle-to-infectivity ratio of particles made in the presence of the *Endo*-1k. HCMV-infected cells were harvested at 96 hpi and sonicated to release virus particles to the culture supernatant. A portion of this supernatant, containing both intracellular and extracellular virions, was treated with DNase I to degrade DNA that was not protected within particles, and then viral DNA in intact particles was released by proteinase K digestion and quantified by real-time PCR. The remaining portion of the supernatant was used to detect the number of plaque forming units (PFUs). In the absence of inhibitors, the particle-to-PFU ratio was ∼122 (Figure 4D, DMSO). Treatment of cells with *Endo*-1k reduced the total particle number by a factor of about 15; however, the treatment reduced the number of infectious virions by a factor of about 350, resulting in a particle-to-PFU ratio of ∼2940. In this experiment, cells received fresh medium containing 10% fetal calf serum at 48 hpi. When the experiment was repeated without changing the medium, *Endo*-1k and Compound 37 completely abolished infectivity of HCMV but only modestly reduced particle number (Figure 4D, right). Apparently, fresh serum diminishes the antiviral activity of the elongase antagonists by providing fatty acids required for efficient production of infectious virus particles.

We conclude that fewer virions of strikingly reduced infectivity are produced in the presence of elongase inhibitors. This might result from the altered fatty acid content of virions, reduced late viral protein accumulation and incorporation into virions, or both.

### HCMV infection robustly increased the cellular and virion content of VLCFAs

**Figure 5:**
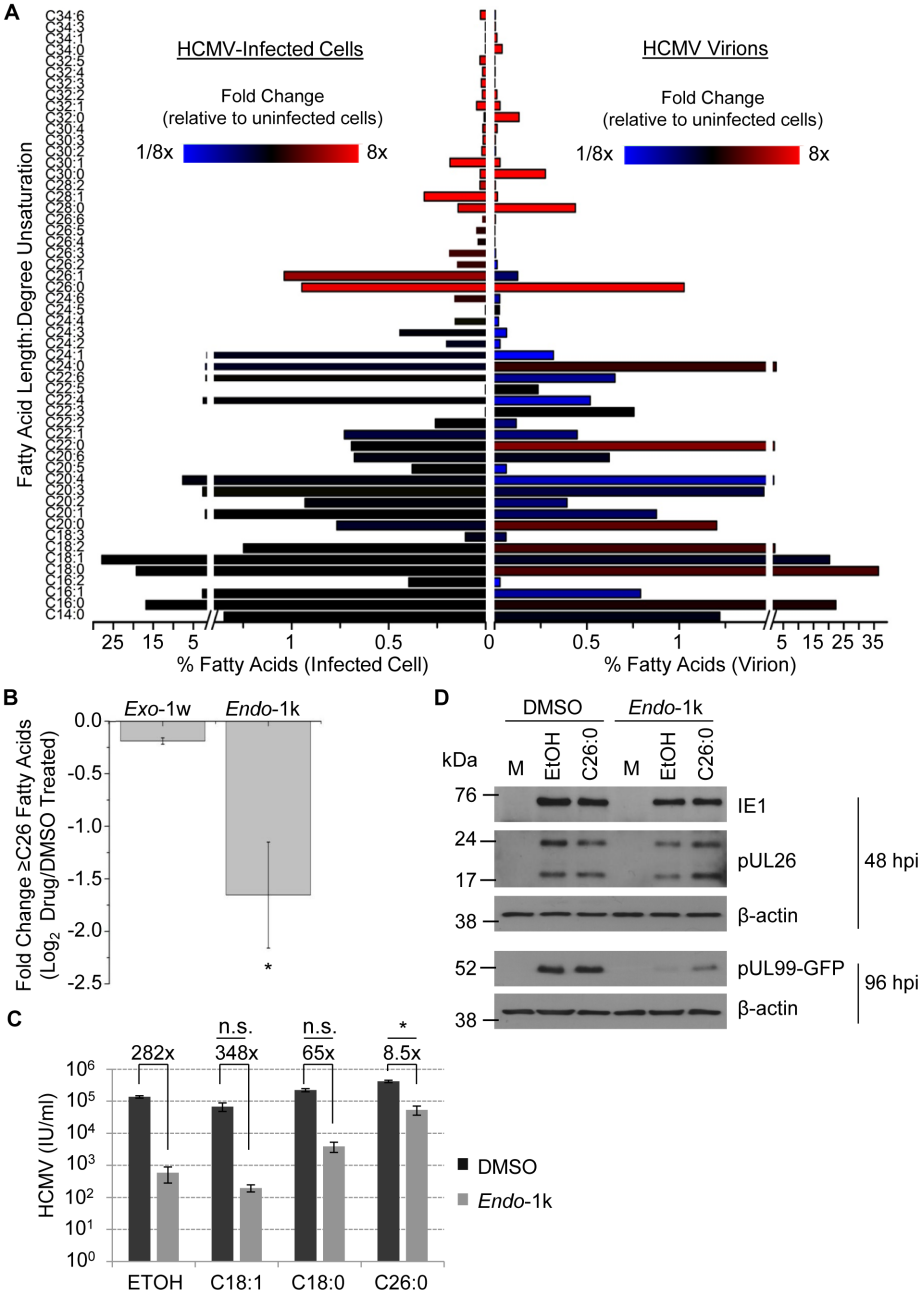
Analysis of the fatty acid content of cells and HCMV virions. **(A)** HCMV infection alters cellular (left side) and virion fatty acids (right side). Fibroblasts were infected with BAD*wt* (3 IU/cell) in serum-free medium. At 72 hpi, total lipids from cells or purified virions were extracted and saponified to release fatty acid tails, which were analyzed by LC-MS. Each fatty acid is identified by its chain length and degree of unsaturation, y-axis. The percent of total signal detected for each fatty acid is represented by bars, x-axis. Bar colors represent the log_2_-fold change over mock-infected cells (black = no change; red = increase; blue = decrease). **(B)** Treatment with elongase inhibitor blocks virus-induced increase in saturated and monounsaturated VLCFAs (≥C26). Fibroblasts were mock infected or infected with BAD*wt* (3 IU/cell) and treated (50 µM) with *Endo*-1k, *Exo*-1w, or DMSO (vehicle). Fresh medium containing the inhibitors was replaced at 48 hpi. Fatty acids were analyzed by LC-MS at 72 hpi. Results report three independent experiments, and error bars represent ±1 SD of the mean. * p<0.05 (t-test, compared to control condition). **(C)** Rescue from drug block with exogenously added fatty acids. BAD*in*UL99GFP-infected fibroblasts (1 IU/cell) were treated with *Endo*-1k (50 µM) or DMSO (vehicle) at 2 hpi. Hexacosanoic acid (C26:0), stearic acid (C18:0), oleic acid (C18:1) or ethanol (ETOH, vehicle) were added (50 µM) with the drug. Cells were harvested at 96 hpi, and the yield of infectious virus was assayed. Results report three independent experiments, and error bars represent ±1 SD of the mean. *p<0.05, **p<0.005, non-significant (n.s.) p>0.05 (t-test, ratio of DMSO/*Endo*-1k in fatty acid treated cells were compared to that of ETOH treated cells). **(D)** The effect of hexacosanoic acid on viral protein accumulation in infected fibroblasts (BAD*in*UL99GFP, 1 IU/cell) treated with *Endo*-1k. Cultures received hexacosanoic acid (C26:0) (50 µM) or ethanol (vehicle for C26:0), simultaneously with drug or DMSO (vehicle for the drug) at 2 hpi. Cells were harvested at indicated times after infection and processed for western blot analysis using antibodies specific for IE1, pUL26, and pUL99. β-actin served as a loading control.

The above results demonstrate that HCMV both up-regulates and depends on VLCFA metabolism. To probe the consequences of this upregulation, we examined the fatty acid composition of mock-infected cells and HCMV-infected cells at 48 hpi. Lipids were extracted from samples and fatty acids were released by saponification and then analyzed by high resolution liquid chromatography mass spectrometry (LC-MS) [20]. While the most prevalent fatty acid tails (16–20 carbons) did not substantially change in abundance, LC-MS analysis revealed a marked increase in VLCFAs that contain ≥26 carbons (Figure 5A, left side). Many of the VLCFA species showed a greater than 8-fold increase compared to uninfected cells.

VLCFAs, especially saturated VLCFAs, were also enriched in the viral envelope, (Figure 5A, right side). For example, C26 fatty acids were increased by a factor of about 8 in both infected cells and virions, and the ratio of saturated C26 fatty acids to their monounsaturated counterparts was about 5-fold higher in virions. The same trend was observed in VLCFAs with increasing tail length, up to C34, the longest class detected. Additionally, shorter chain saturated fatty acids—those that contain 18–24 carbons—were to a lesser extent also enriched. These data demonstrate that lipids with saturated VLCFA tails participate in the assembly of new HCMV progeny, and support the view that inhibition of elongase(s) block viral replication by reducing the accumulation of VLCFAs in the virion envelope.

Next, we analyzed the change in fatty acid tails of lipids in HCMV-infected cells treated with the elongase inhibitor *Endo*-1k (Figure 5B). *Exo*-1w, the inactive derivative of *Endo*-1k, was included as a negative control, and it had only a minor effect when compared to DMSO-treated cells. In contrast, *Endo*-1k substantially reduced the proportion of VLCFA tails ≥C26. To further examine the requirement for elongase activity in the HCMV life cycle, we added hexacosanoic acid (C26:0), stearic acid (C18:0), and oleic acid (C18:1) to HCMV-infected cells in the presence or absence of the elongase inhibitor *Endo*-1k. The saturated VLCFA hexacosanoic acid (C26:0), which is the most abundant VLCFA in the virion envelope among the highly elevated fatty acids in infected cells (Figure 5A), provided almost a complete restoration of HCMV replication and pUL99 accumulation, whereas stearic acid (C18:0) which is a substrate for the synthesis of saturated VLCFAs, had a small restorative effect and the abundant monounsaturated fatty acid oleic acid (18∶1), which is a substrate for monounsaturated VLCFAs that are elevated in the infected cells but not enriched in the virion envelopes, had no effect (Figure 5C). Of note, addition of stearic acid and hexacosanoic acid modestly increased virus yield in the absence of elongase inhibitor, consistent with saturated fatty acids generally supporting viral replication. In line with these results, hexacosanoic acid partially restored the defect in pUL99 accumulation and alleviated the modest reduction in pUL26 levels in *Endo*-1k treated cells but had minimal effect on viral protein expression in untreated cells (Figure 5D). These data confirmed that VLCFA production influences viral early and late protein expression. Overall, these results argue that HCMV depends on elongase activity to produce specific VLCFAs.

HCMV virions acquire their envelope in the cytoplasm in a region adjacent to the nucleus that has been termed the assembly compartment [21]. This compartment is comprised of membranes, virion proteins and cellular proteins derived from the exocytic and endocytic networks, and it is believed to play a critical role in the envelopment process [22], [23], [24]. To test the possibility that elongase activity is required to generate the assembly compartment, we analyzed the compartment using fluorescent confocal microscopy. We monitored the viral structure with the HCMV envelope glycoprotein B (gB, pUL55), which resides in this compartment during late stages of virus replication [21]. As shown in Figure 4C, *Endo*-1k delayed pUL55 accumulation to a limited extent. However, the drug did not prevent pUL55 localization to the assembly compartment and the structures delineated by pUL55 were only slightly reduced in volume due to *Endo*-1k treatment (Figure S2B). These results show that VLCFAs produced by elongases are not required for the assembly compartment to form, although they might nevertheless influence details of its constituents or function. We favor the view that the assembly compartments remain at least partially functional when VLCFA accumulation is blocked, since VLCFAs promote virion infectivity to a greater extent than particle formation (Figure 4D).

### Isotope tracer studies reveal production of VLCFAs via elongation of pre-existing C18 fatty acids

**Figure 6:**
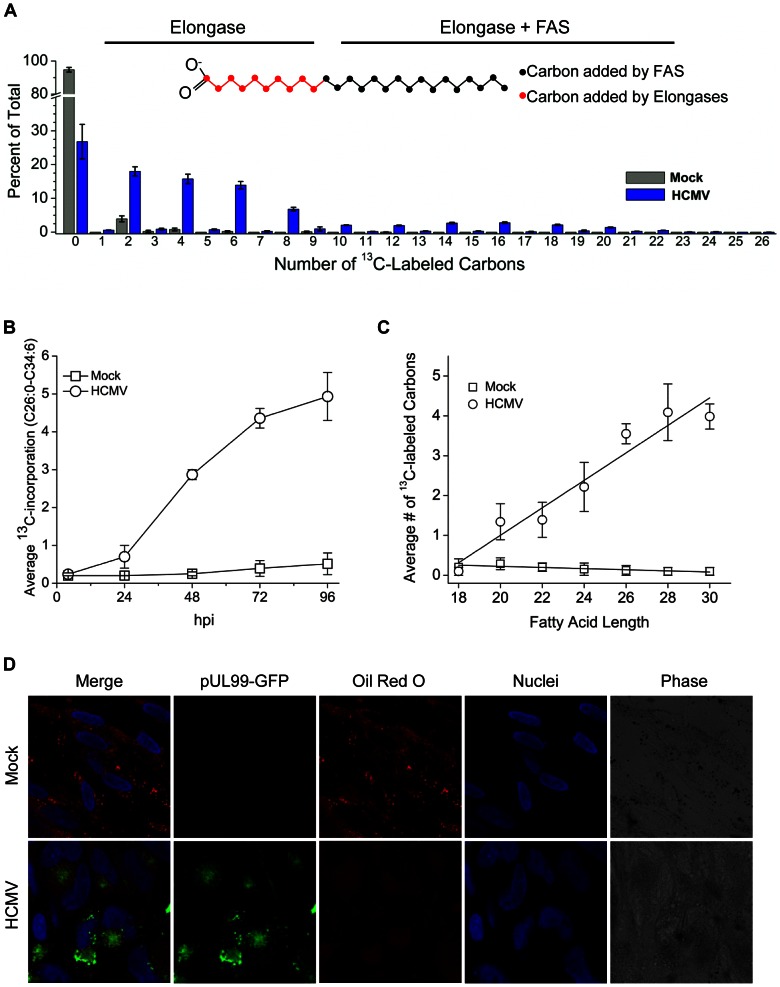
VLCFAs are synthesized from pre-existing fatty acids during HCMV infection. **(A)** Elongation of pre-existing C18 fatty acids. Fibroblasts were either mock-infected or infected with BAD*wt* (3 IU/cell) and feed U-^13^C-glucose starting at 0 hpi. Lipids were extracted at 72 hpi and the ^13^C-labeling of C26:0 fatty acid tails is shown. Inset within the figure is a schematic representation of C26:0 fatty acid. The first 16 carbons are added by fatty acid synthase (FAS, black) and the remaining carbons nearest the carboxylic group are added by elongases (red). **(B)** Kinetic analysis by carbon labeling of ≥C26:0 VLCFAs during HCMV infection. MRC5 cells were infected with BAD*wt* (3 IU/cell), U-^13^C-glucose was added at 0 hpi and saponified fatty acids from total cellular lipids were analyzed by LC-MS at the indicated times. Results report three independent experiments, and error bars represent ±1 SD of the mean. **(C)** The average amount of ^13^C-label incorporated into C18:0-C30:0 at 48 hpi. As in part A, fibroblasts were infected and fed ^13^C-glucose at 0 hpi. The unlabeled fraction (C18:0 and shorter) existed prior infection and the labeled forms (i.e. the longer chains) were formed following infection. **(D)** Analysis of lipid droplets. Fibroblasts were infected with BAD*in*UL99GFP (10 IU/cell). Cells were fixed at 96 hpi and stained with oil red O to visualize lipid droplets (visible in red and phase channels). Infected cells were identified by pUL99-GFP expression (green), and DNA was stained with Hoechst 33258 (blue). The image is representative of >5 different visual fields analyzed in two independent experiments.

To evaluate the source of the VLCFAs produced during HCMV infection, we used ^13^C-glucose to label acetyl-CoA, and thereby malonyl-CoA and resulting fatty acid carbon atoms. The fatty acid labeling was then measured by high resolution LC-MS. In uninfected cells, minimal fatty acid labeling was observed. In contrast, in infected cells, ∼75% of the fatty acids were labeled (Figure 6A). Examination of the fatty acid labeling patterns allowed us to differentiate *de novo* fatty synthesis from elongation. Fatty acid labeling patterns involve a distribution of labeling states, due to the multiple possible routes of synthesizing any given fatty acid (e.g., *de novo* vs. elongation of different pre-existing species) and also the random (i.e., statistical) selection of labeled and unlabeled two-carbon units in synthesizing any particular fatty acid. When fatty acids are made by *de novo* synthesis, the most abundant labeled form occurs when the number of labeled carbon atoms equals the fatty acid chain length multiplied by the fractional acetyl-CoA labeling (50%). For example, if 50% of acetyl-CoA is labeled then the most abundant labeled form of C26 fatty acid made by *de novo* synthesis is that with 12 and 14 labeled carbons (26 carbons added by fatty acid synthase and elongases ×50% acetyl-CoA labeling) [20]. In contrast, when pre-existing fatty acids are elongated, a lesser extent of labeling is observed. For example, when a pre-existing C18 fatty acid is elongated to a C26 fatty acid, the most abundant labeled form has 4 labeled carbon atoms (8 carbon atoms added by elongation ×50% acetyl-CoA labeling). As shown in Figure 6A, in HCMV infected cells the C26:0 fatty acid tail contains predominately 6 or fewer ^13^C labeled carbon atoms demonstrating that elongation activity predominates, with minimal *de novo* VLCFA synthesis.

To determine the kinetics of labeling, uniformly labeled ^13^C-glucose was added at the start of infection (0 hpi) or mock infection. By 24 hpi, labeling of saponified C26–C34 fatty acids was evident in the HCMV-infected cells, and this labeling increased greatly by 48 hpi and slowly thereafter (Figure 6B). By 48 hpi, labeling was >10-fold higher in HCMV-infected than mock-infected cells.

To assess quantitatively the elongation pathway, we evaluated the average extent of ^13^C-labeling as a function of fatty acid chain length (Figure 6C). Starting from C18 fatty acids, which were hardly labeled in both uninfected and infected cells, we observed stepwise increases in labeling with increasing fatty acid chain length in HCMV-infected cells. This suggests that a significant amount of starting material for elongation is pre-existing unlabeled C18 fatty acids. The slope of the plot was 0.7 labeled carbons per added two-carbon unit, which corresponds closely to ∼50% labeling of acetyl-CoA labeling [4]. This implies that 70% of the distinctive VLCFA carbon atoms (beyond C18, i.e. those colored red in Figure 6A) in HCMV-infected cells arise from elongation.

A potential source of the pre-existing C18 fatty acid substrates is lipid droplets, cytoplasmic organelles responsible for the storage of neutral lipids [25]. Certain viruses have been shown to induce lipid droplets [26], [27]. Recently, an increase in lipid droplets was observed during HCMV infection by visualizing the droplets in cells treated with a fluorescent fatty acid analogue that is incorporated into neutral lipids [28]. We monitored the fate of lipid droplets (Figure 6D) by using a histochemical stain, oil red O, that directly stains neutral lipids [29]. In mock-infected fibroblasts lipid droplets were detected as small round structures in the cytoplasm. During HCMV-infection, we observed a loss of lipid droplets in infected cells, consistent with consumption of cellular lipid stores that existed prior to infection to provide fatty acid substrates for elongation. This observation, along with our ^13^C labeling data, indicates that HCMV induces a global re-organization of the cellular lipid environment through modulation of lipid metabolic enzymes.

## Discussion

**Figure 7:**
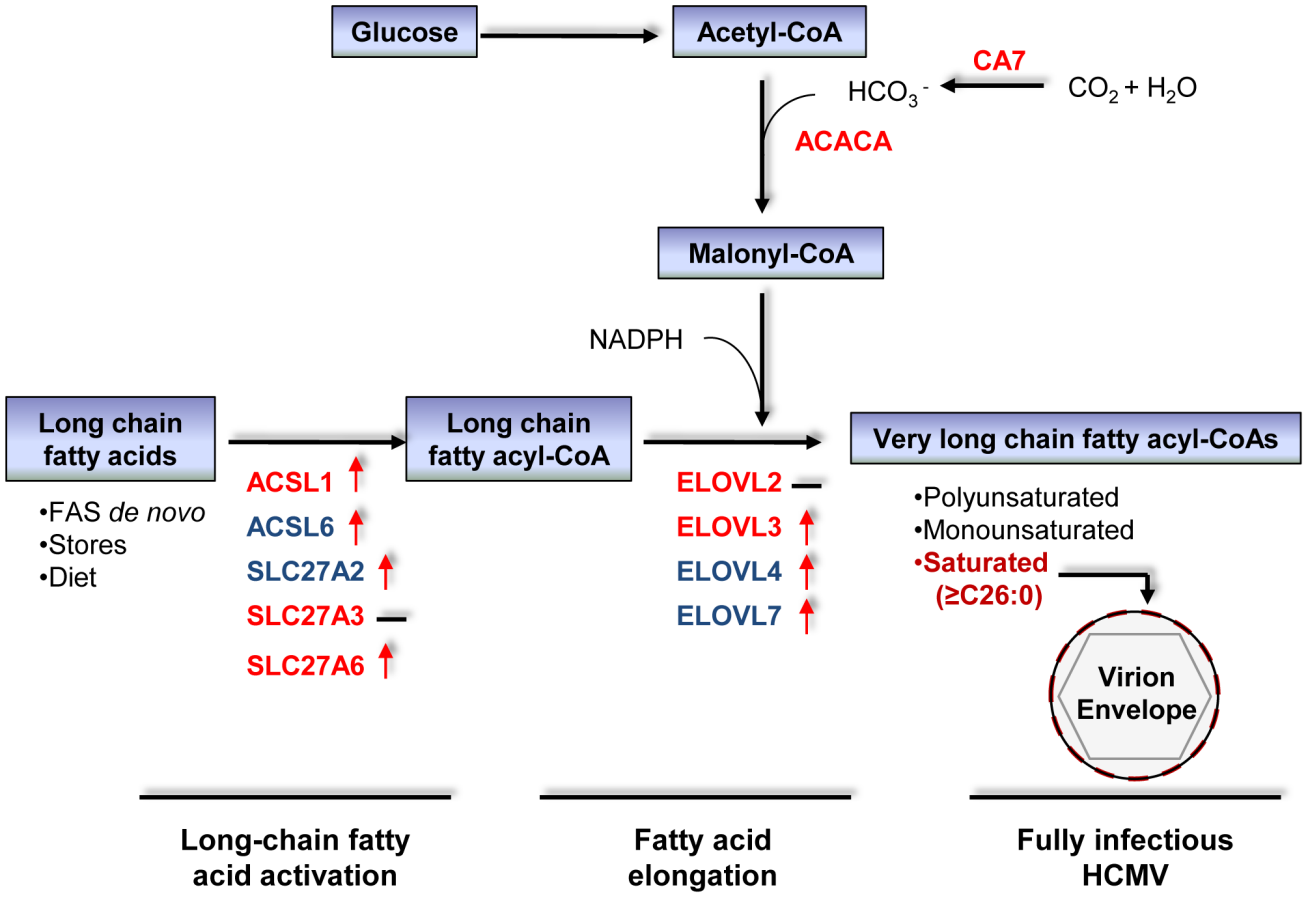
Genes linked to VLCFA synthesis identified by the siRNA screen and RT-qPCR analysis. Genes whose knockdown inhibited HCMV replication are designated red. Blue genes were not assayed in the screen. Red arrows mark genes whose transcript levels are increased after infection and black hyphens mark the genes whose transcript levels do not change more than 3-fold. The expression levels of unmarked genes were not assayed.

We performed an siRNA screen to identify metabolic enzymes required for efficient HCMV replication (Figure 1 and S1; Table S1), and we quantified the expression of fatty acid-related genes following infection (Figure 2 and Table S1 and S2). These analyses highlighted a role for two functionally related protein families during infection: long chain acyl-CoA synthetases and fatty acid elongases (Figure 7). Analysis of saponified fatty acids by LC-MS revealed large increases in C26 and longer species in HCMV-infected cells and virions (Figure 5), and metabolic labeling revealed that elongase activity is dramatically enhanced following infection and accounts for the majority of the observed VLCFAs (Figure 6). The screen also predicted a role during infection for cytosolic carbonic anhydrase 7, which belongs to a family of enzymes that generate bicarbonate [30]. HCO_3_^-^ is consumed in the conversion of acetyl-CoA to malonyl-CoA, a substrate for elongases.

Our mass spectrometry and isotope-tracer data are limited to saponified fatty acids, and thus do not distinguish between different classes of lipids. Two lines of evidence, however, favor the virus primarily depending on phospholipid rather than triglyceride synthesis: virus replication is increased by the knockdown of AGPAT9, which is responsible for triglyceride synthesis [31] (Figure 1B), and lipid droplets, which store neutral lipids, dissociate in HCMV-infected cells (Figure 6D).

The siRNA screen and RNA expression analysis revealed roles for a subset of long chain acyl-CoA synthetases following infection: SLC27A6 and ACSL1 were both siRNA hits and increased at the mRNA level, and SLC27A3 was an siRNA hit but not increased in mRNA abundance. On the other hand, SLC27A2 and ACSL6 were increased at the mRNA level but the former was not identified as an siRNA hit and the latter was not screened in the siRNA assay. Substrate preferences of these acyl-CoA synthetases in human cells are not well characterized, but the mouse ortholog of SLC27A3 can activate saturated LCFAs (C16:0) and VLCFAs (C24:0) [32], which could then serve as substrates for the synthesis of saturated fatty acids ≥C26:0 that accumulate in HCMV virions (Figure 5A). Interestingly, SLC27A3 RNA is elevated in malignant glioma cells, and its knockdown inhibits their proliferation [33]. Recent evidence suggests a link between HCMV and glioblastoma [34], and it is conceivable that modulation of acyl-CoA synthetases by the virus promotes malignancy.

There are seven human elongases, of which ELOVL1, 3, 4, 6 and 7 have been shown to synthesize saturated VLCFAs. One or more of these enzymes is likely responsible for synthesis of the VLCFAs required for viral replication. One of three tested siRNAs against ELOVL3 impaired HCMV replication, and three structurally distinct elongase inhibitors that block ELOVL3 and 6 block the virus. The impact of these inhibitors on ELOVL7, whose mRNA is most strongly upregulated by HCMV, has not been determined. Thus, further experiments are needed to definitely determine the set of elongases required to produce infectious HCMV progeny.

Why does HCMV infection require elongases and ACSLs? Like ACC inhibition, ACSL inhibition blocks HCMV replication without impairing late protein expression. This is consistent with VLCFAs playing an essential role in either particle assembly or viral entry into host cells in the next replication cycle. Such a role is also consistent with our observation that elongase inhibition results in particles with very low infectivity (Figure 4D). The blockade of late protein synthesis by elongase inhibitors raises the possibility of a role for VLCFAs also earlier in the HCMV replication cycle, but is not sufficient to establish such a role: elongase inhibition could lead to accumulation of malonyl-CoA, which can be cytotoxic [35] and thereby indirectly impair the viral lifecycle.

Additional insights into the role of VLCFA come from analyses of the virion envelope. In addition to VLCFAs, the viral envelope is enriched for saturated fatty acids (Figure 5). The higher levels of saturated fatty acid tails in the viral envelope, relative to infected host cells, are consistent with the virus acquiring its envelope from a specialized subcellular compartment such as the HCMV assembly compartment. Alternatively, there could be enrichment for certain lipids during the viral budding process, perhaps because these lipids favor membrane curvature or other physical features necessary to bud virions. For example, lipid containing C26:0 fatty acid has been shown to stabilize membrane curvature [36]. Further, C26:0 modulates the packing of phospholipids in model membranes and promotes lipid phase separation (“raft formation”) [37]. These physical effects can modulate protein localization. For example, C26:0 alters localization of plasma membrane ATPase in yeast, independent of the identity of the head group to which it is attached [38]. Thus, saturated VLCFA might be critical for organizing HCMV envelope proteins, with the proper protein and lipid composition to produce infectious viral progeny.

Why has HCMV evolved to rely on the synthesis and remodeling of lipids for the production of infectious progeny? One possible explanation relates to latency. HCMV actively replicates in many cell types, but remains quiescent in monocytes and reactivates to produce infectious progeny only when monocytes differentiate into macrophages [39], [40]. Interestingly, a dramatic increase in fatty acid synthesis is observed when monocytes differentiate [41]. This raises the possibility that HCMV has evolved to remain latent in cells unable to synthesize VLCFAs in response to infection, and to reactivate when host cell acquire this metabolic capacity.

## Materials and Methods

### Cells, viruses and drug treatments

Human MRC5 fibroblasts (ATCC) were cultured in medium (DMEM) containing 10% fetal calf serum (FCS) and 4.5 g/L glucose. BAD*wt* [42] and BAD*in*UL99GFP [43] were produced from bacterial artificial chromosome (BAC) clones of the HCMV AD169 strain in MRC5 cells, and titers were determined by TCID_50_ or fluorescent focus assays [44]. Both viruses replicate with wild-type kinetics.

Triacsin C was from Sigma; and *Endo*-1k, compound 37, and (*S*)-1y as well as the inactive derivatives *Exo*-1w and *(R)*-1y were kind gifts from Dr. J. Kim (Kadmon Corporation). Stock solutions of the compounds were freshly prepared for each experiment at 10 mM in DMSO. To assess HCMV growth in the presence of inhibitors, cells were infected at a multiplicity of 1 infectious unit (IU)/cell, and 2 h later the inoculum was replaced with medium containing 10% FCS and drug or DMSO alone. Unless otherwise indicated, the medium with inhibitors was replenished at 48 hpi. For metabolite rescue experiments, unesterified oleic acid (Sigma), stearic acid (Sigma), and hexacosanoic acid (Cayman) solutions were freshly prepared at 10 mM in ethanol. Fatty acids were diluted into medium containing 10% FCS. Control wells received the same volume of ethanol diluted in medium. Cells were scraped and sonicated at 96 hpi, and titers were determined on MRC5 cells. Cell viability was assessed by CellTiter 96 AQueous One Solution assay (Promega). The effect of drugs on cell morphology and pUL99-GFP expression was analyzed at 96 hpi.

### siRNA screen

MRC5 cells were seeded in 96-well plates at a density of ∼7,500 cells per well. Two days later, cells were washed with Opti-MEM (Invitrogen) and transfected with siRNAs from the Mission (Sigma) siRNA Metabolism and Cell Traffic Panel (217 targets) and Transferase Panel (184 targets) using Oligofectamine (Invitrogen). Each 96-well plate contained siRNAs specific for cellular PLK1 (siPLK1) and siNT (siRNA Universal Negative Control #1, Sigma) as negative controls, and three different siRNAs targeting cellular kinase VPS34 (VPS34_1–3) plus an siRNA targeting the HCMV IE2 RNA (siIE2) [45] were positive controls. The design of the plates is indicated in Figure S1A and sequences of siRNAs are listed in Table S3. The final concentration of duplex siRNAs was 167 nM per well in 60 µl Opti-MEM. At 16 h after transfection, 60 µl DMEM containing 20% serum was added to the wells, and 8 h later, cells were infected with BAD*in*UL99GFP in 100 µl fresh DMEM containing 10% serum. The multiplicity of infection (0.5 IU/cell) and time of harvest (96 hpi) were selected so that 50 µl of the supernatant would contain ∼2,000 infectious particles per well in the negative controls. This generated a linear dynamic range for the assay ranging from an ∼3.75-fold increase to a >8-fold decrease in virus titers (Figure S1B). The hits identified by the screen all fell within this range.

To control for edge effects caused by the prolonged incubation of plates and to enable plate-to-plate comparisons, we performed a two-step normalization procedure. First, the data were normalized to the median value of each row with coordinates row A and columns 2 to 11 (A-2–11), B-, C-, D-, E-, F- and G-3–10, and H-2–11. Since edge effects were observed in outer wells including the column 1, 2, 11, and 12, rows B–G (B-1 – G-1, B-2 – G-2, B-11 – G-11, and B-12 – G-12) the values in these wells were normalized to the column median. This strategy yielded a mean of 0.995±0.156 (standard deviation, SD) for 72 siPLK1-specific siRNAs placed as internal controls in the screen. Next, the fold change in virus yield relative to the mean of these negative controls was calculated and log_2_-transformed. An siRNA was categorized as a potential hit if it altered the virus yield by 3 times more than the SD of negative controls.

To evaluate the efficiency of the siRNA screening protocol, Z′-factors, 1 – (3× SD of positive control + 3× SD of negative control)/|mean of positive control – mean of negative control|, were calculated [46]. This provides a measure of signal window and Z′>0 indicates a dependable assay, when calculated from positive controls that are similar in strength to the hits [47]. The control siRNAs targeting host RNA, siVPS34_1, 2 and 3, generated Z′-factors of 0.16, 0.03, and 0.03, respectively.

### Analysis of nucleic acids and proteins

To quantify RNA levels using commercial PCR arrays, total RNA was extracted from mock and HCMV-infected cells (10 IU/cell) at 48 hpi using RNeasy Kit (Qiagen), reverse transcribed using the RT^2^ First Strand Kit and amplified using Human Fatty Acid Metabolism and Human Adipogenesis PCR arrays with RT^2^ SYBR Green/ROX qPCR Master Mix (SABiosciences). Transcript levels were normalized by comparison to the arithmetic mean of four different housekeeping cDNAs (B2M, HPRT, RPL13A, and GAPDH) and fold differences between mock and virus-infected samples were calculated. To quantify elongase RNAs, cDNA was synthesized from total RNA using Multiscribe RT (Applied Biosystems). Primers specific to seven different elongase genes were designed using QuantPrime software [48] and used in qPCR reactions with SYBR Green PCR master mix (Applied Biosystems). Results were normalized to GAPDH. Sequences of elongase and GAPDH primers are listed in Table S3.

To assess an appropriate threshold for distinguishing significantly altered transcript levels during HCMV infection, we determined a null distribution of fold change by conducting pair wise comparisons of fold change in mock- and virus-infected replicates. When a cut-off threshold of ≥3-fold change was applied only 1.9% false positives were detected.

Virus particles were quantified by qPCR analysis of DNase I-resistant viral DNA in virion preparations [49]. Purified BAC*wt* DNA was used to generate standard DNA concentration curves.

Protein accumulation was analyzed by western blot assay [50] by using antibodies specific for ACSL1(Cell Signaling), ELOVL3 (Sigma), IE1 (1B12) [51], IE2 (3A9) [52], pUL55 (Abcam), pUL83 (8F5) [53], pUL99 (10B4-29) [54], pUL44 (Virusys) and pUL26 (7H-19) [55]. β-actin or α-tubulin levels were detected as a loading control using anti-β-actin (Abcam) or anti-tubulin antibody (Sigma). Protein bands were visualized using ECL reagent (Amersham), and band intensities were quantified using Image J software.

### Analysis of fatty acids

For analysis of fatty acids from cellular lipids, confluent MRC5 cells were serum-starved for 24 h prior to infection (3 IU/cell). Cells were maintained in serum-free medium until the time of lipid extraction. Isotopic metabolic labeling experiments were performed by growing cells in serum-free DMEM that contained fully labeled U-^13^C-glucose (Cambridge Isotopes) following viral infection. Results were corrected for naturally occurring carbon 13 [4], [20]. When the lipid content of cells treated with elongase inhibitor or DMSO was examined, the cell growth conditions were as described above; however, the serum-free medium and drug/vehicle control were replenished 48 hpi. Analysis of virion fatty acids was performed on particles purified from the supernatant of infected fibroblasts grown in serum-free medium. Virus particles were pelletted through a 20% sorbitol cushion and then subjected to centrifugation in two sequential sodium tartrate gradients [56]. Extraction of lipids from cells and purified virions was as described [57]. Briefly, cells and virions were lysed using cold 50% methanol containing 0.05 M HCl. Lipids were extracted by adding chloroform at final ratio of 2∶1, with vortexing and centrifugation. The chloroform layer was transferred to a glass vial and the extraction was repeated to ensure complete isolation of lipids. All steps were carried out at 4°C. Next, lipids were dried under nitrogen gas. The fatty acid tails were liberated from the lipid head group by saponification after resuspending the dried lipids in 1 ml 90% methanol 0.3 M KOH and heating to 80°C for 1 h. Afterwards the samples were neutralized using formic acid. Next an equal volume of hexane was added to the samples and briefly vortexed. The hexane layer containing fatty acids were transferred to a new glass vial and the hexane extraction was repeated. After drying using a nitrogen stream, each sample was resuspended in 1 ml (for cells from one 60 cm culture dish) or 0.5 ml (for virions from 6 roller bottles) chloroform∶methanol∶water (1∶1∶0.3). Samples for mass spectrometry analysis were analyzed in duplicate and each experiment was repeated at least three times.

Fatty acids were analyzed by LC/MS [20]. The samples containing the isolated fatty acids were diluted 4–5-fold prior to 10 µl being loaded onto a Luna C8 reversed-phase column (Phenomemex 00F-4248-B0) held at 25°C. LC employed two buffers: buffer A was 97∶3 water∶methanol with 10 mM tributylamine and 15 mM acetic acid (pH 4.5), and buffer B was 100% methanol [58]. A gradient from 80% B to 99% B was run linearly for 20 min, held at 99% B for an additional 20 min, and from 99% back to 80% B linearly over 1 min and then held at 80% B for a final 10 min. The flow rate at each step was 0.2 ml/min. The LC step was carried out with an inline orbitrap mass spectrometer (Thermo Exactive) running in negative mode. After column separation the sample was vaporized by electrospray ionization with a 3 kV spray voltage. Other MS parameters were: capillary temperature, 325°C; capillary voltage, −25 V; and a scan range of 200–400 m/z for the first 20 min and 300–575 m/z for 30 additional min. MS data was analyzed using MAVEN: Metabolomic Analysis and Visualization Engine [59]. Additional programs used to analyze and visualize the results included Excel (Microsoft), MATLAB (The MathWorks), PRISM (GraphPad), and Origin (OriginLabs).

### Fluorescent analysis of lipid droplets and the viral assembly compartment

Lipid droplets were assayed at 96 h after infection (3 IU/cell) or mock infection by using oil red O [60]. Nuclei were marked by staining DNA with Hoechst 33258 (Invitrogen).

To monitor the volume of the viral assembly compartment, BAD*in*UL99GFP-infected cells (0.1 IU/cell) were fixed at 72 hpi and stained with antibody specific for HCMV glycoprotein B (gB, pUL55) [61] and secondary antibodies conjugated to Alexa-594 (Molecular Probes). For volume analysis, z-stack images (0.29 µm) were collected using an SP5 LSM confocal microscope (Leica Microsystems) for the entire individual cells and analyzed using Velocity 4.0 software (Improvision). The pUL55 containing assembly zones were detected using an intensity cut-off of 45 units and volumes smaller than 100 µm^3^ were eliminated to remove the pUL55-associated cytoplasmic structures outside the viral assembly zone.

### Test of statistical significance

All p-values were calculated using two-tailed, unpaired t-test.

### Accession numbers

Acetyl-CoA acyltransferase 1 (ACAA1): P09110. Acetyl-CoA carboxylase 1(ACACA): Q13085. Acyl-CoA synthetase long-chain 1 (ACSL1): P33121. Acyl-CoA synthetase long-chain 6 (ACSL6): Q9UKU0. 1-acylglycerol-3-phosphate O-acyltransferase 9 (AGPAT9): Q53EU6. Carbonic anhydrase 7 (CA7): P43166. Elongation of very long chain fatty acids protein 1 (ELOVL1): Q9BW60. Elongation of very long chain fatty acids protein 2 (ELOVL2): Q9NXB9. Elongation of very long chain fatty acids protein 3 (ELOVL3): Q9HB03. Elongation of very long chain fatty acids protein 4 (ELOVL4): Q9GZR5. Elongation of very long chain fatty acids protein 5 (ELOVL5): Q9NYP7. Elongation of very long chain fatty acids protein 6 (ELOVL6): Q9H5J4. Elongation of very long chain fatty acids protein 7 (ELOVL7): A1L3X0. Fatty acid synthase (FAS): P49327. Phosphatidylinositol 3-kinase catalytic subunit type 3 (PIK3C3, VPS34): Q8NEB9. Polo-like kinase 1 (PLK1): P53350. Solute carrier family 27 member 2 (SLC27A2): O14975. Solute carrier family 27 member 3 (SLC27A3): Q5K4L6. Solute carrier family 27 member 6 (SLC27A6): Q9Y2P4. HCMV immediate early protein 1 (IE1): P13202. HCMV immediate early protein 2 (IE2): P19893. HCMV tegument protein UL26 (pUL26): P16762. HCMV DNA polymerase processivity factor (pUL44): P16790. HCMV envelope glycoprotein B (pUL55): P06473. HCMV 65 kDa phosphoprotein UL83 (pUL83): P06725. HCMV tegument protein UL99 (pUL99): P13200.

## Supporting Information

Figure S1 **siRNA screen.** **(A)** Schematic representation of the siRNA screen. Locations of control siRNAs and empty wells are color coded. **(B)** siRNAs specific for IE2 block the accumulation of the viral gene product. Fibroblasts were transfected with either non-targeting siRNAs (siNT) or IE2 siRNAs (siIE2) infected (0.5 IU/cell) 24 h later. Cells were harvested at 96 hpi and analyzed by western blot by using an antibody detecting IE2. α-tubulin served as a loading control. **(C)** pUL99 accumulation is blocked in cells transfected with siRNAs targeting viral IE2. Fibroblasts were transfected with either non-targeting siRNAs (siNT) or IE2 siRNAs (siIE2) and infected (0.5 IU/cell) 24 h later. At 96 hpi images were captured by fluorescent microscopy to visualize cells expressing pUL99-GFP. **(D)** Linear dynamic range of the screen. Fibroblasts were infected with indicated amount of infectious virus in 96-well plates. At 24 hpi, cells were fixed and number of infected cells in each well was quantified. The multiplicity of infection used in the screen yields ∼2000 infectious virus particles (marked by dashed line) at 96 hpi in wells containing control siRNAs that do not influence virus yield. Arrows above or below the dashed line indicate that an increase (3.75 fold) or decrease (8 fold) in virus yield within these limits are in the range over which the yield is linear with number of infected cells.
(TIF)

Figure S2 **Analysis of the viral assembly compartment in cells treated with** ***Endo*** **-1k and hexacosanoic acid.** Fibroblasts were infected with BAD*in*UL99GFP (0.1 IU/cell) and treated with *Endo*-1k, C26:0, or DMSO at 2 hpi as indicated. Fresh medium containing the inhibitors was replaced at 48 hpi. Cells were fixed at 72 hpi and stained with antibody specific for pUL55 and Hoechst 33258 to visualize DNA. **(A)** Immunofluorescent analysis of viral proteins in the assembly compartment. Z-stack images (0.29 µm) were collected and collapsed down into maximum intensity projections to visualize pUL99-GFP (green), pUL55 (red), and DNA (blue). The z-stacks were used in the 3D reconstruction of assembly zones using pUL55 fluorescence (Merge – pUL55 3D). **(B)** Estimated volume of the viral assembly compartment based on pUL55 fluorescence. More than 40 cells with well-isolated assembly compartments were analyzed for each treatment. Bars indicate average volume of assembly compartments. Error bars represent standard error of the mean. **p<0.005, non-significant (n.s.) p>0.05 (t-test, compared to control condition).
(TIF)

Table S1 **siRNA Screen.** Fibroblasts were transfected with siRNAs and infected with BAD*in*UL99GFP (0.5 IU/cell) 24 h later. HCMV yield in the supernatant was determined at 96 hpi. Three siRNAs were tested for each target, and their effect on HCMV yield was quantified and log_2_-transformed. siRNAs that inhibited HCMV replication are designated in red type and siRNAs that elevated virus production are designated in green type. Fold change in RNA levels (HCMV/mock) of the genes assayed in the screen is indicated. (Results are from data in Table S2).
(XLS)

Table S2 **Expression levels of genes related to lipid and fatty acid metabolism during HCMV infection.** Fibroblasts were mock-infected or infected with BAD*in*UL99GFP (10 IU/cell). RNA was analyzed at 48 hpi by RT-qPCR. Results were normalized to the expression level of housekeeping genes and presented as fold change (HCMV/mock).
(XLS)

Table S3 **siRNA and primer sequences used in this study.**
(XLS)
