# Supplementary figures and images for: Saturated Very Long Chain Fatty Acids Are Required for the Production of Infectious Human Cytomegalovirus Progeny
Source: PLoS Pathog. 2013 May 16;9(5):e1003333. doi: 10.1371/journal.ppat.1003333 (PMC3656100; doi:10.1371/journal.ppat.1003333)

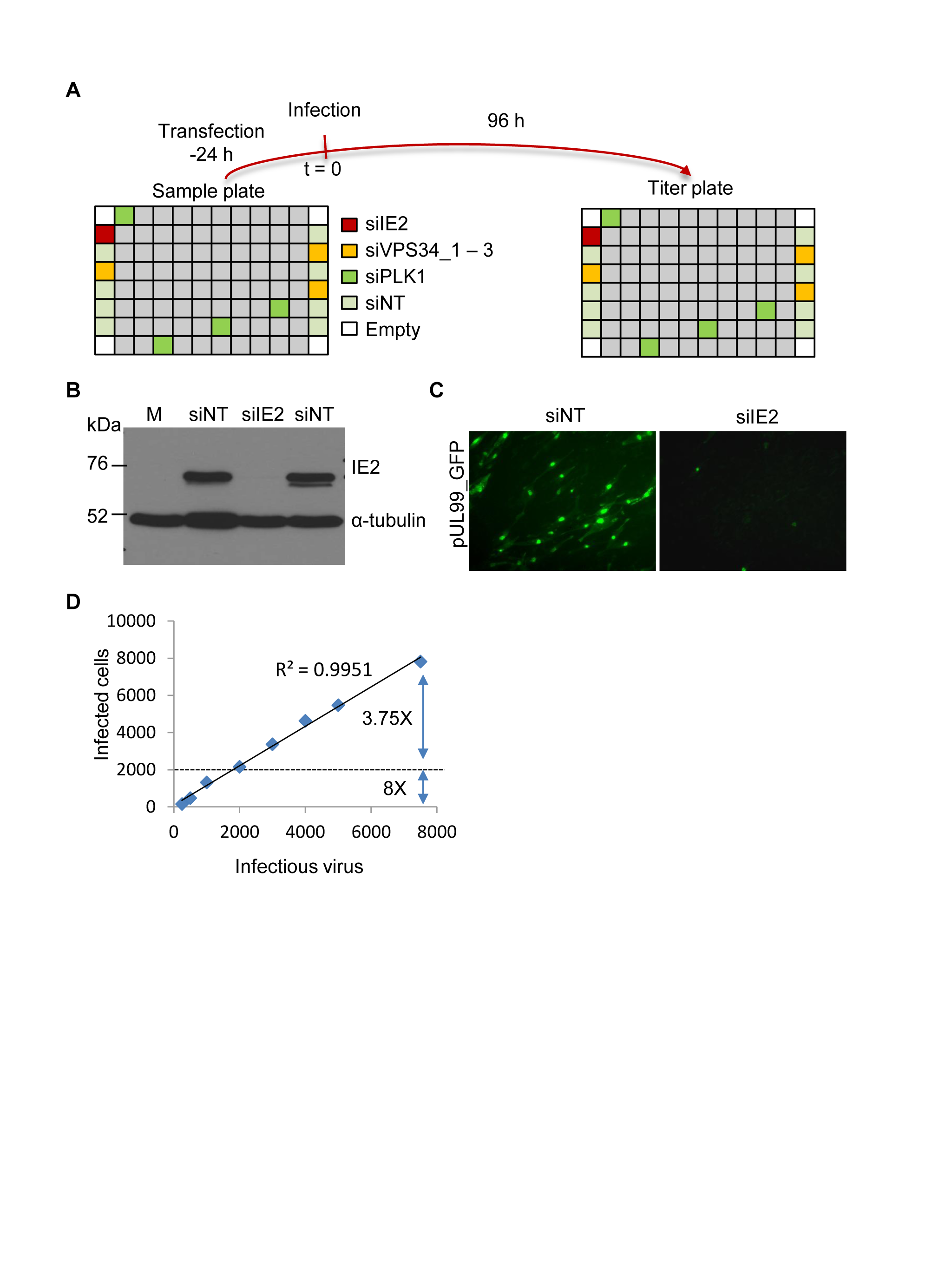

Supplement: Figure S1 — siRNA screen. (A) Schematic representation of the siRNA screen. Locations of control siRNAs and empty wells are color coded. (B) siRNAs specific for IE2 block the accumulation of the viral gene product. Fibroblasts were transfected with either non-targeting siRNAs (siNT) or IE2 siRNAs (siIE2) infected (0.5 IU/cell) 24 h later. Cells were harvested at 96 hpi and analyzed by western blot by using an antibody detecting IE2. α-tubulin served as a loading control. (C) pUL99 accumulation is blocked in cells transfected with siRNAs targeting viral IE2. Fibroblasts were transfected with either non-targeting siRNAs (siNT) or IE2 siRNAs (siIE2) and infected (0.5 IU/cell) 24 h later. At 96 hpi images were captured by fluorescent microscopy to visualize cells expressing pUL99-GFP. (D) Linear dynamic range of the screen. Fibroblasts were infected with indicated amount of infectious virus in 96-well plates. At 24 hpi, cells were fixed and number of infected cells in each well was quantified. The multiplicity of infection used in the screen yields ∼2000 infectious virus particles (marked by dashed line) at 96 hpi in wells containing control siRNAs that do not influence virus yield. Arrows above or below the dashed line indicate that an increase (3.75 fold) or decrease (8 fold) in virus yield within these limits are in the range over which the yield is linear with number of infected cells. (TIF) [file ppat.1003333.s001.tif]

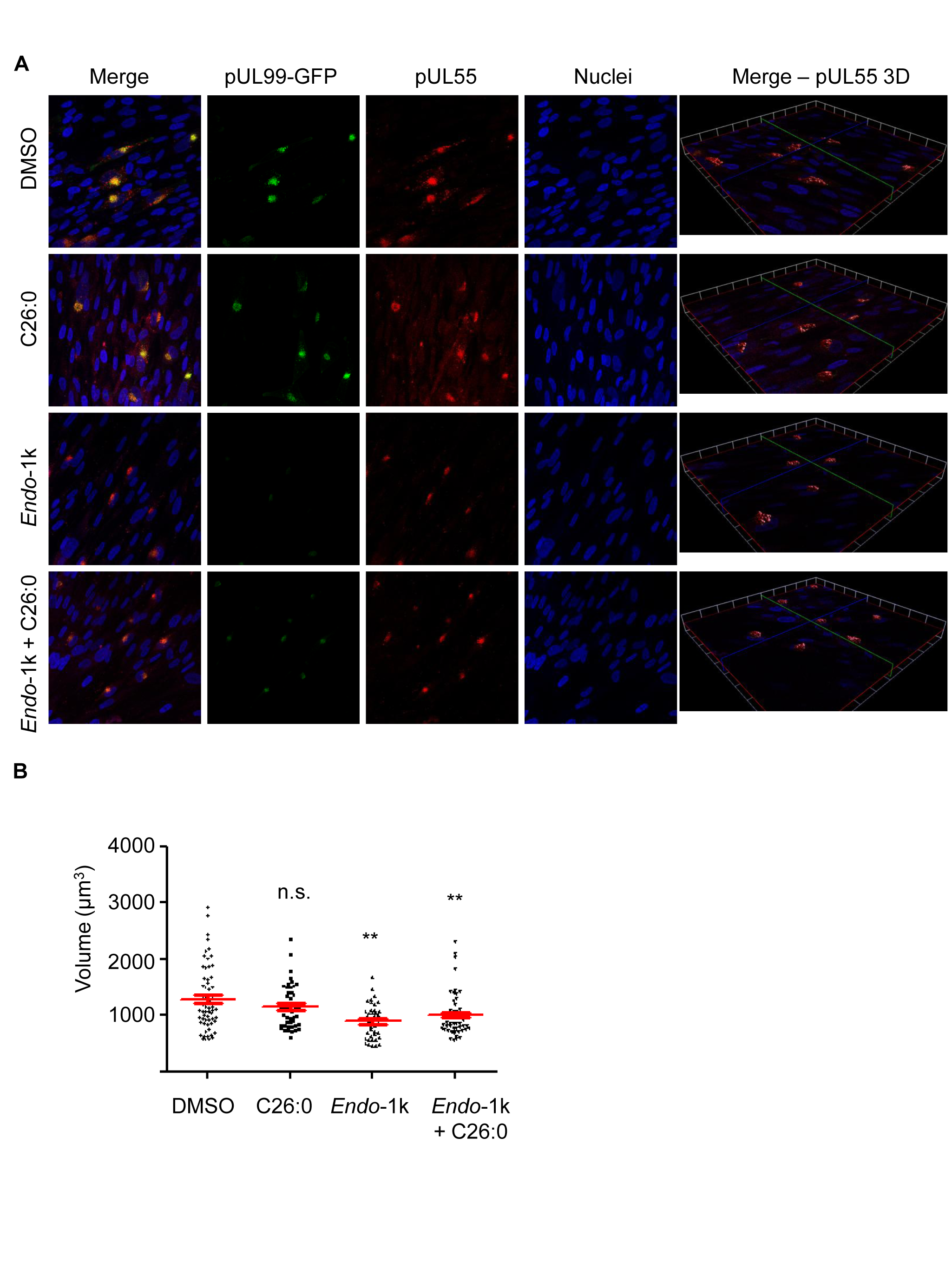

Supplement: Figure S2 — Analysis of the viral assembly compartment in cells treated with Endo -1k and hexacosanoic acid. Fibroblasts were infected with BADinUL99GFP (0.1 IU/cell) and treated with Endo-1k, C26:0, or DMSO at 2 hpi as indicated. Fresh medium containing the inhibitors was replaced at 48 hpi. Cells were fixed at 72 hpi and stained with antibody specific for pUL55 and Hoechst 33258 to visualize DNA. (A) Immunofluorescent analysis of viral proteins in the assembly compartment. Z-stack images (0.29 µm) were collected and collapsed down into maximum intensity projections to visualize pUL99-GFP (green), pUL55 (red), and DNA (blue). The z-stacks were used in the 3D reconstruction of assembly zones using pUL55 fluorescence (Merge – pUL55 3D). (B) Estimated volume of the viral assembly compartment based on pUL55 fluorescence. More than 40 cells with well-isolated assembly compartments were analyzed for each treatment. Bars indicate average volume of assembly compartments. Error bars represent standard error of the mean. **p<0.005, non-significant (n.s.) p>0.05 (t-test, compared to control condition). (TIF) [file ppat.1003333.s002.tif]
